# Immunoliposomes for detection of rupture-prone intracranial aneurysms

**DOI:** 10.1007/s00701-023-05770-9

**Published:** 2023-09-26

**Authors:** Behnam Rezai Jahromi, Vladimir Zamotin, Christian Code, Eliisa Netti, Martina B. Lorey, Kari Alitalo, Katariina Öörni, Aki Laakso, Riikka Tulamo, Mika Niemelä

**Affiliations:** 1 Department of Neurosurgery, Helsinki University Hospital and University of Helsinki, Topeliuksenkatu 5, 00260 Helsinki, Finland; 2 Neurosurgery Research Group, Biomedicum Helsinki, Helsinki, Finland; 3 PHYLIFE: Physical Life Science, Department of Physics, Chemistry and Pharmacy, University of Southern Denmark, Odense M, Odense, Denmark; 4 Wihuri Research Institute, Biomedicum Helsinki, Haartmaninkatu 8, Helsinki, Finland; 5 Department of Vascular Surgery, Helsinki University Hospital and University of Helsinki, Helsinki, Finland

**Keywords:** Cerebral aneurysm, Imaging, Inflammation, Intracranial aneurysm, Liposome, Subarachnoid hemorrhage

## Abstract

**Background:**

It is estimated that significant (3.2%) of population carries intracranial aneurysm (IA). An increasing number of imaging studies have caused that the chance of finding an incidental aneurysm is becoming more common. Since IA rupture causes subarachnoidal hemorrhage (SAH) and have significant mortality and morbidity prophylactic treatment should be considered when IA is detected. The benefit and risk of treatment of IA is based on epidemiological estimate which takes account patient and aneurysm characteristics. However we know that aneurysm rupture is biological process where inflammation of aneurysm wall is actively leading to degeneration of aneurysm wall and finally weakens it until it bursts. Until now, there have not been imaging method to detect inflammatory process of aneurysm wall

**Methods:**

We created targeting immunoliposome for use in the imaging of aneurysm. Immunoliposome comprises antibodies against at least one vascular inflammatory marker associated with aneurysm inflammation and a label and/or a contrast agent.

**Results:**

Histological analysis of IAs where immunoliposome comprises antibodies against vascular inflammation with a label shows promising results for selectively detecting aneurysms inflammation. In magnetic resonance imaging (MRI) we were able to detect immunoliposomes carrying gadolinium.

**Conclusion:**

Our work opens a new avenue for using contrast labeled immunoliposomes for detecting rupture-prone aneurysms. Immunoliposomes can cary gadolinium and selectively bind to inflammatory section of aneurysm that can be detected with MRI. Further research is needed to develop immunoliposomes to be used with MRI in humans to target treatment to those patients who benefit from it the most.

**Supplementary Information:**

The online version contains supplementary material available at 10.1007/s00701-023-05770-9.

## Introduction

With the increasing imaging of brain angioarchitecture, incidental intracranial aneurysms (IAs) are found more often. Around 2–3% of the world population carries IAs. In case of a rupture, the aneurysm causes a subarachnoid hemorrhage (SAH). Despite the current treatment methods, approximately half of the patients with SAH die, and half of the survivors are left with a neurological disability [2].

Fortunately, the majority of IAs never rupture. Current evaluation of aneurysm rupture risk depends on patient related risk factors, aneurysm morphology in imaging studies, and location. Since endovascular and microneurosurgical aneurysm treatment carries risks, selection of which patients with unruptured IAs should be treated has remained problematic.

The IA wall undergoes dynamic regeneration and degeneration highly dependent on inflammation, which either stabilizes the aneurysm wall or eventually leads to IA wall tear leading to SAH [9].

Current imaging lacks methods to differentiate between stable aneurysms and aneurysms at rupture risk. Several studies have aimed at imaging inflammation of IA to detect future rupture risk [4]. However, most of these studies lack sensitivity and thus the overall aim to detect a rupture-prone aneurysm has yet to be established.

Our aim was to create a gadolinium loaded immunoliposome with the ability to bind to an epitope expressed on the rupture-prone aneurysm wall which is detectable via magnetic resonance imaging (MRI).

Immunoliposomes are lipid nanoparticles with an antibody attached to the surface and they are used for diagnostic and therapeutic applications [7, 8]. Immunoliposomes with antibodies against inflammatory proteins on the aneurysm wall can be targeted to specific sites in the arterial wall. Furthermore, immunoliposomes can be loaded with drugs, dyes, or contrast agents and can be made to have a size of 100 nm making them small enough to get to the aneurysm target in the vasculature but to have enough internal capacity to achieve signal enhancement in medical imaging [8].

Here we describe a proof-of-concept study of development of such gadolinium-containing immunoliposome with conjugated antibodies on its surface enabling its binding on a selected cellular target with signal enhancement in MRI in vitro.

## Materials and methods

### Materials

The detailed consistency of immunoliposomes and preparation of them are described in supplement material 1 and 2.

### Negative control

For negative control a lipid formulation without maleimide group has been used. Then, corresponding antibody has been added and this mixture underwent same steps of the conjugation protocol described above. Thus, we ensure that antibody in immunoliposomes and mixture of antibody and control liposomes subjected to the same actions.

### Tissue sections

The human intracranial aneurysm tissue was resected after clipping of an aneurysm, snap frozen and stored in −80 °C. The collection of the tissue was approved by the local ethical committee. The fresh frozen human tonsil tissue, resected during tonsillectomy, was collected and stored in −80 °C as anonymous control tissue. The frozen tissue embedded in Tissue-Tek (Sakura Finetek, Netherlands) and cryosectioned at 4 μm. The sections were air dried for 30 min and kept in −20 °C until the time of staining.

### Cell cultures

Human coronary artery endothelial cells (HCAEC, PromoCell, Heidelberg, Germany) were cultured in Endothelial cell basal medium MV (PromoCell) supplemented with the Supplement Pack Endothelial Cell GM MV (PromoCell) comprising fetal calf serum, endothelial cell growth supplement, recombinant human epidermal growth factor, heparin, and hydrocortisone; as well as 100 U/ml penicillin and 100 μg/mL streptomycin (Lonza, Basel, Switzerland) under standard cell culture conditions (37 °C, 5% CO_2_). For microscopy, 6 × 10^4^ cells were seeded into each well of Lab-Tek II 4-well Chamber slides (Nunc A/S, Roskilde, Denmark), cultured until they reached 80–95% confluency, and fixed with 4% PFA for 15 min and kept in PBS until the time of staining.

Human coronary artery smooth muscle cells (CaSMC, Clonetics, Lonza) were cultured under standard cell culture conditions (37 °C, 5% CO_2_) in Smooth Muscle cell Basal Medium (Clonetics Lonza) supplemented with SmGm-2 SingleQuots supplement pack containing epidermal growth factor, fibroblast growth factor, insulin, gentamicin sulfate, and fetal bovine serum; as well as 100 U/ml penicillin and 100 μg/ml streptomycin (Lonza). For microscopy, 2.5 × 10^4^ cells were seeded into each well of Lab-Tek II 4-well Chamber slides (Nunc), cultured until they reached 80–95% confluency, and fixed with 4% PFA for 15 min and kept in PBS until the time of staining.

### Immunofluorescence staining of tissue sections and cell cultures

The tissue sections and cultured cells on slides were stained with immunofluorescence staining methods for aSMA and aCD31 to serve positive controls for the binding of immunoliposomes. The slides were fixed by incubation with 4% PFA or ice-cold acetone for 5 min and rinsed in PBS, followed by serum-blocking with 5% of normalhorse serum (NHS) in PBS for 30 min and incubated with primary antibody of mouse anti-human aSMA (clone 1A4, Invitrogen, Carlsbad, USA; with dilution 1:200) or mouse anti-human CD31 (clone JC70A, DAKO, Santa-Clara, USA; with dilution 1:200) diluted in PBS, for 30 min at room temperature (RT) solely or followed by +6 C overnight. The slides were rinsed in PBS and F(ab’)2 fragment of goat anti-mouse IgG Alexa fluor 488 (Invitrogen, Carlsbad, USA; with dilution 1:40) was incubated for 30 min in dark at the RT, rinsed with PBS. DAPI to stain nuclei (Invitrogen, with dilution 1:40) was added and incubated for 15 min. At last, slides were embedded in fluorescence mounting media, Fluoromount-G (Invitrogen, Carlsbad, USA), covered with cover slip and stored in dark at +6 C until the time of microscopy.

**Fig. 1:**
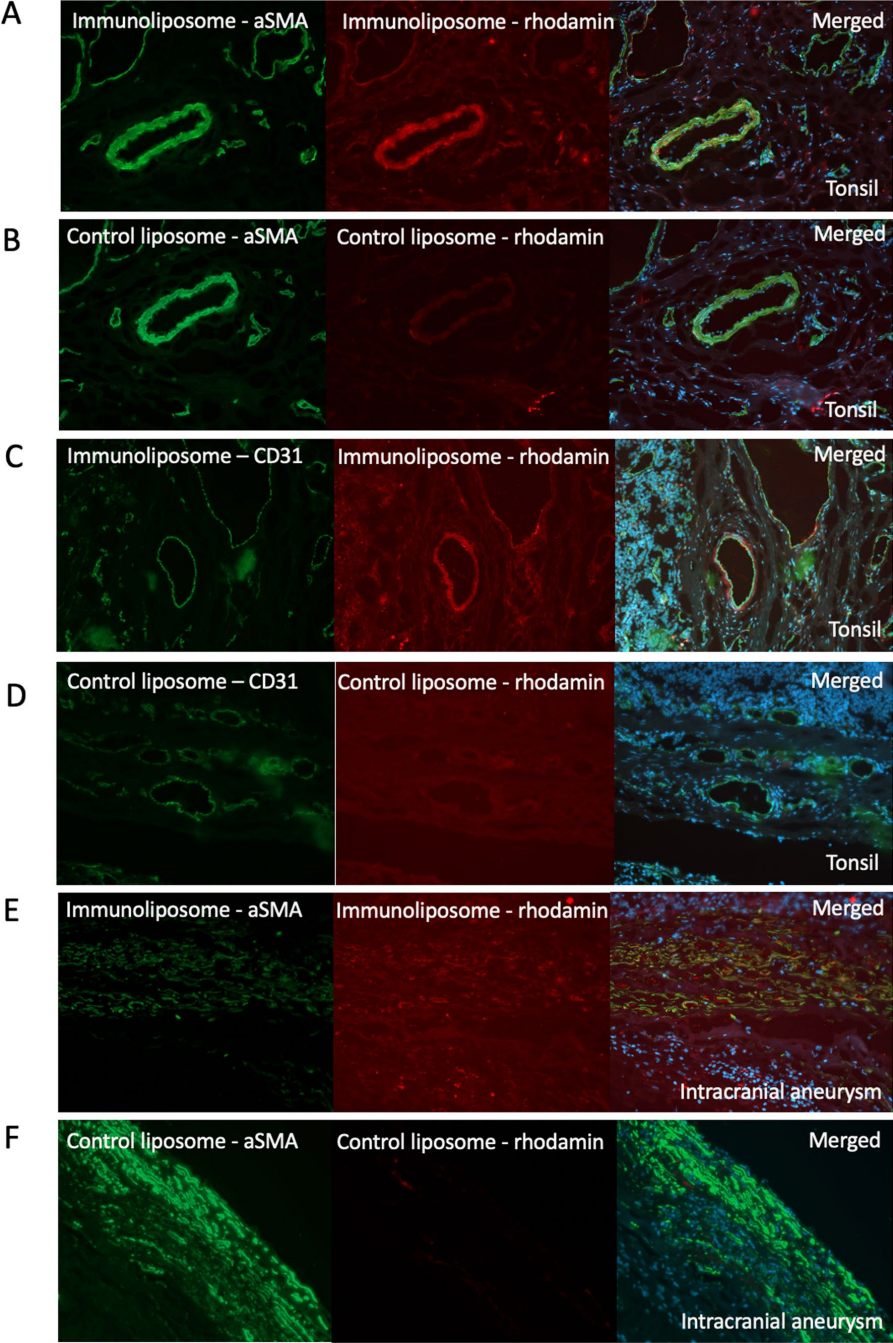
Stainings on tonsil (**A**–**D**) or intracranial aneurysm (**E**–**F**) for aSMA- or CD31-conjugated immunoliposomes and unconjugated control liposomes, where, in each panel, the first image shows the immunostaining for antibody (green), the second image shows rhodamine-conjugated lipids (red), and the last image merges the channels with staining for DAPI (blue) for nuclei

For the confirmation of the successful immunostaining for aSMA, the positive signal was detected in the vascular smooth muscle cells in the walls of the small arteries and veins in the tonsil sections, in the intramural aSMA positive cells located in the aneurysm sample, and in intracellular actin structures of the cultured smooth muscle cells, whereas other structures and cells remained negative for aSMA (Fig. 1). For the confirmation of the successful staining for CD31, the positive signal was detected in the luminal lining of a cell monolayer, i.e., in the endothelium, of the small arteries and veins in the tonsil sections, in asimilar endothelial lining of the luminal border of the aneurysm sample, and in the cell membrane of the cultured endothelial cells, whereas other structures and cells remained negative for CD31 (Fig. 1). The negative controls with omitted primary antibodies remained negative.

### Immunoliposome binding on tissue sections and cell cultures

The tissue sections and cultured cells on slides were tested for immunoliposome binding with immunoliposomes conjugated with the anti-human aSMA and anti-human CD31 primary antibodies. Liposomes in mixture with unconjugated antibodies served as negative controls. Immunoliposomes and liposomes were prepared both with lipids conjugated with rhodamine and without rhodamine.

All tissue sections were fixed. For fixed tissue sections and cells, the slides were first incubated with 4% PFA or ice-cold acetone for 5 min and rinsed in PBS. All slides were serum-blocked with 5% of NHS for 30 min and incubated either with immunoliposome or control liposome diluted in PBS in 1 mM of lipid concentration for 2 h at RT solely or followed by +6 C overnight. The slides were rinsed in PBS and Alexa fluor 488 (Invitrogen, with dilution 1:40) was incubated for 30 min in dark at the RT, rinsed with PBS and DAPI(Invitrogen, with dilution 1:40) to stain nuclei was added and incubated for 15 min. At last, slides were embedded in fluorescence mounting media (DAKO), covered with cover slip, and stored in dark at +6 C until the time of microscopy. For the negative control of these stainings the primary antibody was omitted.

### Microscopy

The tissue sections and cells cultured on slides were analyzed under ObserverZ.1 fluorescence microscope (Zeiss AG, Oberkochen, Germany) at ×10, ×20, and ×40 magnifications, photographed with Axiocam camera (Zeiss AG) and further viewed with ZEN2 software (Zeiss AG). The images were analyzed for the location of the positive signal in the tissue slides or cultured cells.

### MR imaging

For a confirmation of the MR detection of the gadolinium contained immunoliposomes (Gd-immunoliposomes) conjugated with aSMA antibody, both immunoliposomes loaded with Gd chelate DOTAREM and liposomal formulation with Gd-conjugated lipid were imaged. Immunoliposomes were diluted in PBS in varying Gd concentrations from 50 μg/mL to 5 mg/ml and imaged in a phantom made by casting agar gel prepared in tap water in a mold with wells. A series of corresponding concentrations of gadolinium preparation diluted PBS served as positive control and PBS without gadolinium as negative control. The imaging was done under 3 Tesla MR scanner (Siemens) with T1/2/ sequences.

### Characterization of immunoliposomes in vitro

- Thermal stability of liposomes

Temperature transition of lipid formulation is defined mostly by length of lipid acyl chain. In liposomes prepared they are palmitoyl chain in DPPC with phase transition at 41 °C and stearoyl chains in pegylated DSPE with phase transition at 55 °C. As immunoliposomes supposed to operate in human body we tested their thermal stability by using fluorescent dye, carboxyfluorescein, loaded inside liposomes. The dye loaded in liposomes results in self-quenching effect and concentration of entrapped molecules so high that excitation light cannot cause emission from all dye molecules. However, if integrity of lipid bilayer is disturbed, the leakage of the dye outside causes enhancement of fluorescent signal. This effect can be observed either by heating liposomes and reaching phase transition temperature of lipid mixture or by adding detergent and destroying liposome shell. Supplementary figure 1 A demonstrates thermal stability of immunoliposomes at temperatures closed to physiological by measuring CF self-quenching effect and Supplementary figure 1 B shows that immunoliposomes preserve their integrity during and after conjugation protocol by using same self-quenching effect upon addition 10% of Triton-X.

b) Size control of immunoliposomes

Size and polydispersity of prepared immunoliposomes have been controlled after every step conjugation protocol. Besides, both parameters may indirectly serve as a sign of liposome integrity. Supplementary figure 2. A-D shows that average size has been remained during preparation around 100 nm. However, polydispersity index slightly bigger for immunoliposomes compared to controlled liposomes which can be explained by attachment of conjugated antibodies.

c) Justification of conjugation protocol by using small model protein lysozyme and IgG

Hen lysozyme was used as a model protein to establish the protocol and to characterize conjugation of the protein to the produced targeting liposomes. Lipid to which protein is conjugated has a molecular weight around 3 kDa and lysozyme’s molecular weight is around 14.5 kDa. Thus, lipid-protein conjugated structure can be resolved by SDS-gel electrophoresis as a second band slightly upper to monomeric protein (Supplementary figure 3 A). Unfortunately, SDS-gel cannot be applied to directly confirm antibody conjugated to lipid as the resolution in the upper part of SDS-gel is not enough. Nevertheless, rough estimation of antibody conjugated structures can be assayed by SDS-gel by comparison of pellets and supernatants of the same sample after ultracentrifugation at 100,000 G. Precipitation of monomeric IgG at such a gravity force is little, therefore double washing by ultracentrifugation minimizes contribution of IgG to pellet fraction. Since intensity of gel bands corresponds to amounts of protein, their analysis by ImageJ software gives us an efficiency of conjugation around 20% (Supplementary figure 3 B). Amounts of antibodies used in our preparation of immunoliposomes are relatively small, so methods described in this section are not applicable to unequivocally confirm successful conjugation. Given, both aSMA and aCD31 antibodies used in immunoliposomes are based on IgG scaffold, we may expect that conjugation protocol is complied. Also, we can admit conjugation efficiency as 20% based on the results obtained for the IgG case.

## Results

### Immunoliposomes on tissue sections

The immunoliposomes conjugated with anti-human aSMA antibodies showed binding on vascular smooth muscle cells (VSMCs) in tissue sections of tonsil and intracranial aneurysms (Fig. 1A, B, E, F). The immunostaining of the immunoliposome conjugated antibodies was observed in the same locations as the corresponding immunostainings for aSMA (not shown). The rhodamine-conjugated lipids were seen in similar locations as aSMA staining, but a faint signal was also detected in areas negative for aSMA. The signal was stronger in sections stained with immunoliposomes than with control liposomes suggesting antibody-targeted binding of immunoliposomes, but not excluding the possibility of nonselective binding of liposomes to all cells.

The immunoliposomes conjugated with anti-human CD31 antibodies bound to vascular endothelium in the tonsil sections. Similarly, to aSMA-conjugated immunoliposomes, the signal originating from the rhodamine-conjugated lipids was detected in selected CD31-negative cells and faintly with the control liposomes (Fig. 1C, D).

### Immunoliposomes on cell cultures

**Fig. 2:**
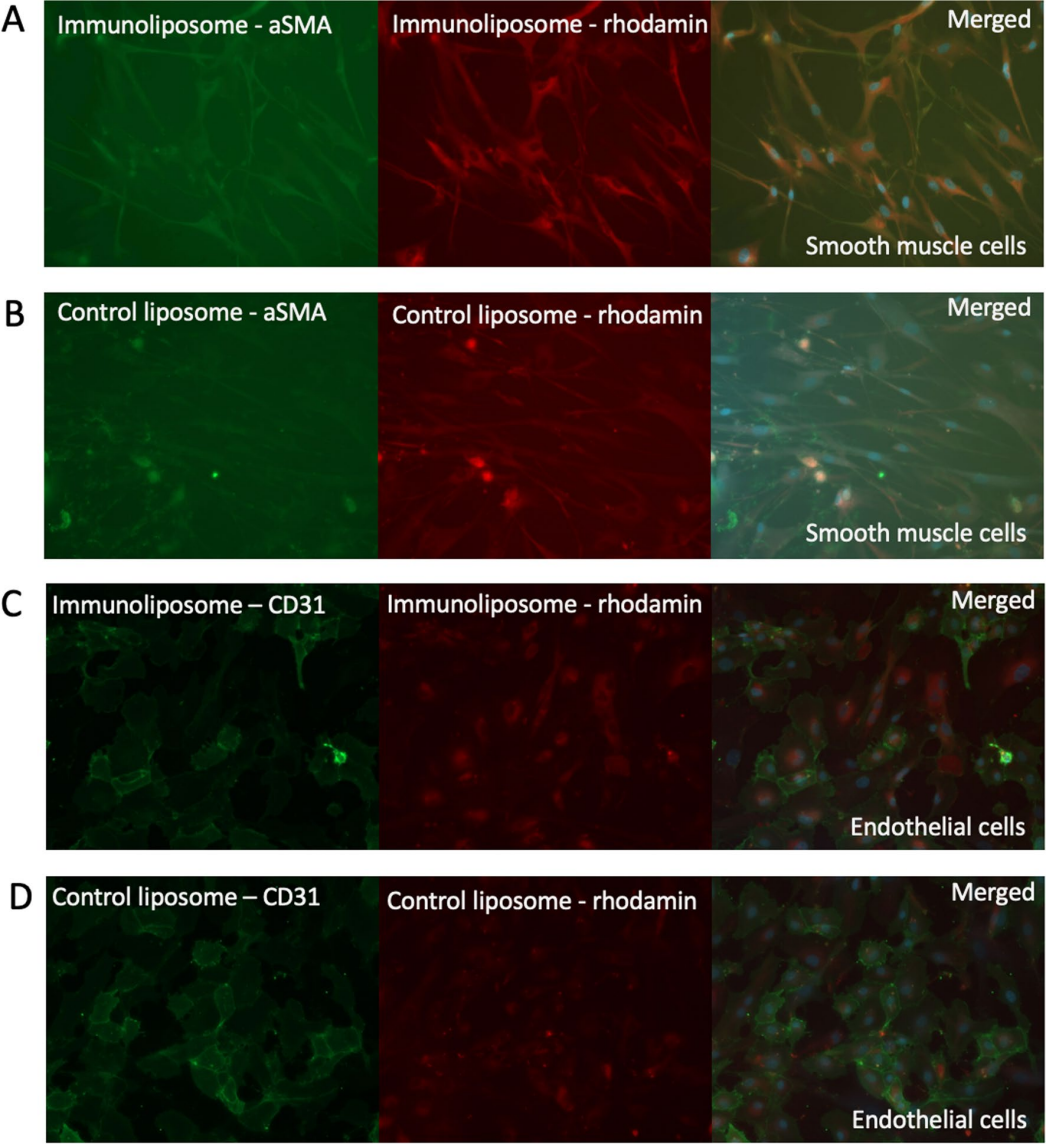
Stainings for aSMA- or CD31-conjugated immunoliposomes and unconjugated control liposomes on cultured smooth muscle cells (**A**–**B**) and endothelial cells (**C**–**D**), where, in each panel, the first image shows the immunostaining for antibody (green), the second image shows rhodamine-conjugated lipids (red), and the last image merges the channels with staining for DAPI (blue) for nuclei

The immunoliposomes conjugated with anti-human aSMA antibodies showed intracellular binding in fixed cultured VSMCs (Fig. 2A). The staining pattern was similar to immunostaining for aSMA (not shown). The positive signal originating from the rhodamine-conjugated lipids was seen in similar locations as aSMA. However, also unconjugated liposomes prepared with rhodamine-conjugated lipids resulted in some intracellular signal suggesting internalization of the lipids or liposomes (Fig. 2B).

The immunoliposomes conjugated with anti-human CD31 antibodies, and the control liposomes, showed analogous binding on the fixed cultured endothelial cells (ECs) as was seen with aSMA-conjugated immunoliposomes on cultured VSMCs (Fig. 2C, D). In ECs, the positive staining for CD31 was detected on the cell membrane.

The immunoliposomes conjugated with anti-human CD31 antibodies were also tested on unfixed ECs. The selective binding of CD31 antibody was detected on ECs kept at +4 °C before staining (Fig. 2). When cells were kept in cell culture conditions until staining, almost no binding was seen (data not shown). The unfixed ECs that showed a positive signal were shrunken with some blebbing of the cell membrane reflecting probable cell death. Thus, most likely the binding relates to the expression and exposition of the epitopes on the cell membrane and the cell membrane permeability. The signal for rhodamine-conjugated liposomes showed a similar but fainter signal pattern than with fixed cells.

### Gadolinium-conjugated immunoliposomes in MR imaging

**Fig. 3:**
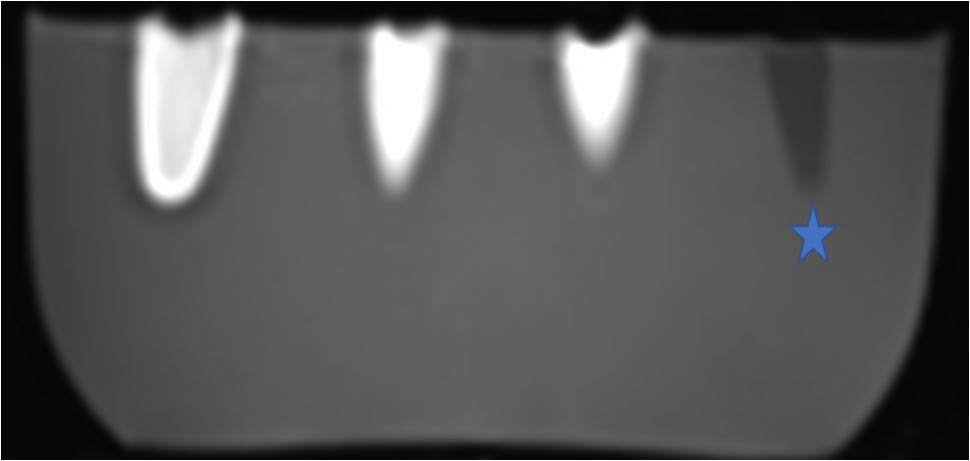
MRI (3 Tesla) of gadolinium-containing immunoliposomes at different concentrations in vitro with T1 sequence seen as white area in phantom, and control (tap water) seen as grey (star)

The gadolinium-containing immunoliposomes conjugated with aSMA antibody, both encapsulated with gadolinium and by using the gadolinium-conjugated lipids, showed a positive signal in MR imaging (3 Tesla Siemens) in T1 sequence even at low concentrations, suggesting a potential for detectability of the immunoliposomes also in vivo, whereas the negative controls remained negative (Fig. 3). When the concentration of gadolinium was increased, a corresponding signal void was detected (data not shown).

## Discussion

Increased availability of MRI has improved the detection of incidental IAs. Unfortunately, clinicians have no tools to differentiate between the stable IAs and those carrying the risk of rupture. Thus, there is an emerging need to target the rupture-prone aneurysms for their invasive treatment. The current imaging methods are restricted to analysis of aneurysm morphology and in some extent to gadolinium enhancement in MRI probably reflecting an increased aneurysm wall permeability [2, 4].

In this proof-of-concept study, we describe the development of a gadolinium-containing immunoliposome with conjugated antibodies on its surface enabling its binding on a selected cellular target with signal enhancement in MRI in vitro with a potential to be used in imaging of IAs with the ultimate goal of detection of rupture-prone aneurysms. Immunoliposomes loaded with gadolinium and targeted to dysfunctional endothelium through antibody binding have been studied both in vitro [5] and in vivo in mice [1]. We studied the immunoliposomes in vitro both in human tissue samples and in cultured human VSMCs and ECs. We showed that the immunoliposomes bound to their targets in vitro, and immunoliposomes, both encapsulated with gadolinium and prepared with gadolinium-conjugated lipids were detectable in MRI in vitro even with very low concentrations of gadolinium. The rhodamine-conjugated lipids were added to visualize the location of lipids with a fluorescent microscope. A slight background staining from cells or tissue negative for the aimed epitope was detected. This is most likely due to lipid-lipid interactions with cell membranes leading to internalization of lipids as described earlier [3]. Increasing the cholesterol content in liposomes can increase the stability of the liposomes and decrease the internalization [6]. However, we were not completely able to abolish the internalization with this adjustment. The phenomenon was less detected, the more intact the cells were. Since the targeted cells and tissues in rupture-prone IAs are already under stress, the detected phenomenon is hypothesized to aid in targeting the molecule in inflamed tissues.

Immunoliposomes can be challenging to create and to make them stable enough. They should simultaneously carry both the antibody to attach to the specific epitope expressed in IA and the chelated gadolinium to illuminate the attachment via MRI as well as avoid activating immunological and coagulation systems. Immunoliposomes can be functionalized by adding one or several external antibodies on the outer leaflet using well documented techniques [8]. They can be made stable, non-toxic with a long half-life, uniform, and small so they get to the intended target in vivo [6]. Thus, immunoliposomes can be used as theragnostic devices offering both a diagnostic and potential targeted therapeutic (Strayoula et al 2008) for intracranial aneurysms.

## Conclusion

Gadolinium-containing immunoliposome for detection of rupture-prone intracranial aneurysms in MRI is a fascinating new method that has now been demonstrated in vitro in this proof-of-concept study. We have shown that such liposomes can be created, that the liposomes bind selectively to their target cellular structures, with minor background binding through probable lipid-lipid interactions. These liposomes can be encapsulated or conjugated with gadolinium and can be used in imaging enhancement in vitro. However, future studies are warranted to prove the usefulness of these liposomes in vivo and further in real-life differentiation between stable and rupture-prone aneurysms. Although development of immunoliposome for detecting rupture-prone IAs is a work in progress it shows promises to solve a serious clinical dilemma—to treat or to not treat.

### Supplementary information

ESM 1 (PDF 773 kb)

ESM 2 (DOCX 15 kb)

## Data Availability

In the manuscript provided.
