# Supplementary material for: Immunoliposomes for detection of rupture-prone intracranial aneurysms
Source: Acta Neurochir (Wien). 2023 Sep 26;165(11):3353–60. doi: 10.1007/s00701-023-05770-9 (PMC10624708; doi:10.1007/s00701-023-05770-9)
Supplement: Supplementary file 1 — (PDF 773 kb) [file 701_2023_5770_MOESM1_ESM.pdf]

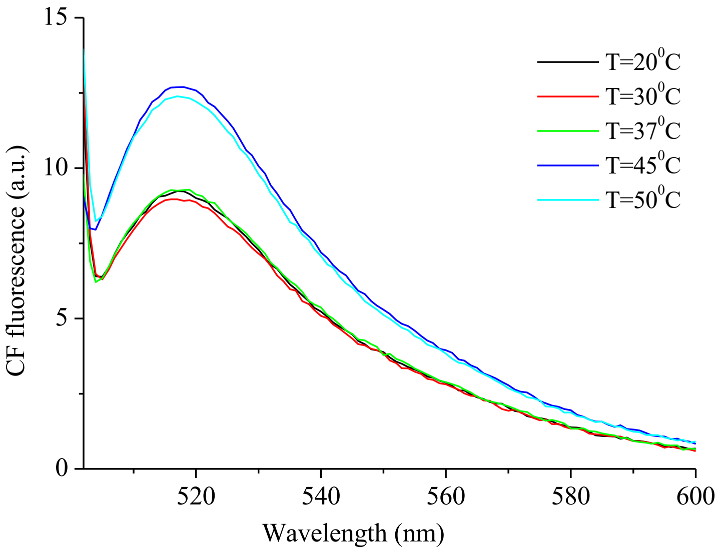

Supplementary Figure 1-A. Thermal stabiltiy of liposomes with encapuslated carboxyfluorescein (CF) measured based on self-quenching of CF and its release upon heating

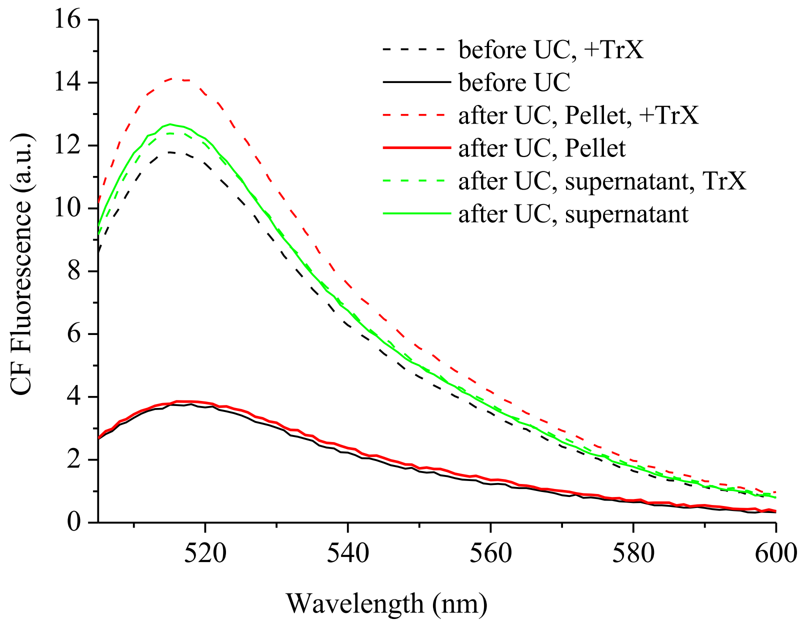

Supplementary Figure 1-B. Integrity of liposomes before and after ultracentrofugation with encapuslat carboxyfluorescein (CF) measured based on self-quenching of CF and its release after addition detergent Triton-X

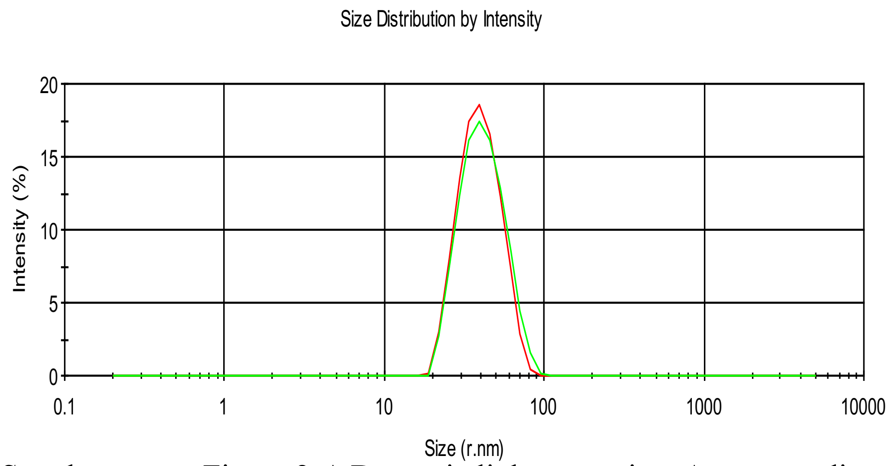

Supplementary Figure 2-A Dynamic light scattering. Average radius of immunoliposome and
control liposome formulations after extrusion

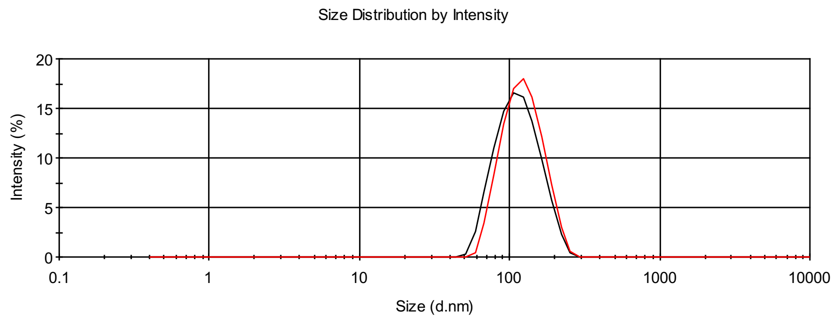

Supplementary Figure 2-B Dynamic light scattering. Average diameter of aSMA-immunoliposomes (black line)
and control liposome with aSMA (red line) after ultracentrifugation (pellet fraction)

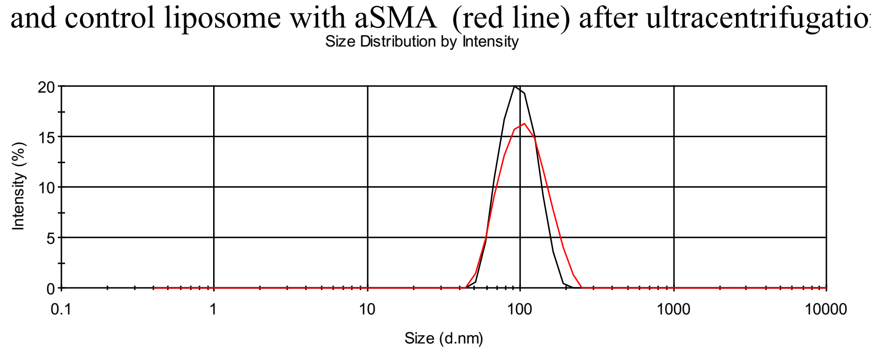

Supplementary Figure 2-C Dynamic light scattering. Average diameter of aCD31-immunoliposomes (black line)
and control liposome with aCD31 (red line) after ultracentrifugation (pellet fraction)

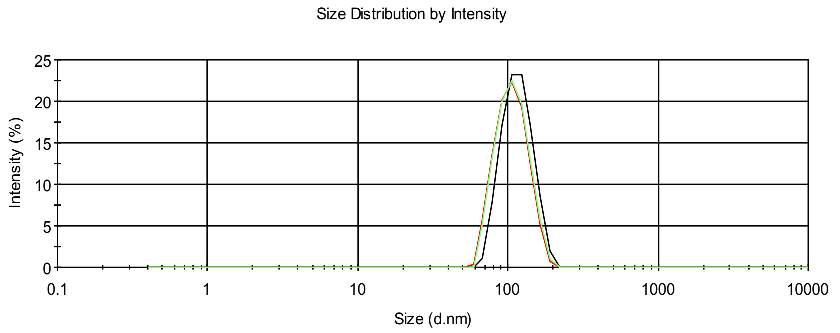

Supplementary Figure 2-D Dynamic light scattering. Average diameter of Gd-immunoliposome formulations:
black line, DOTAREM liposome; green line, GD-DTPA is 5mol%; red line, Gd-DTPA is 10mol%

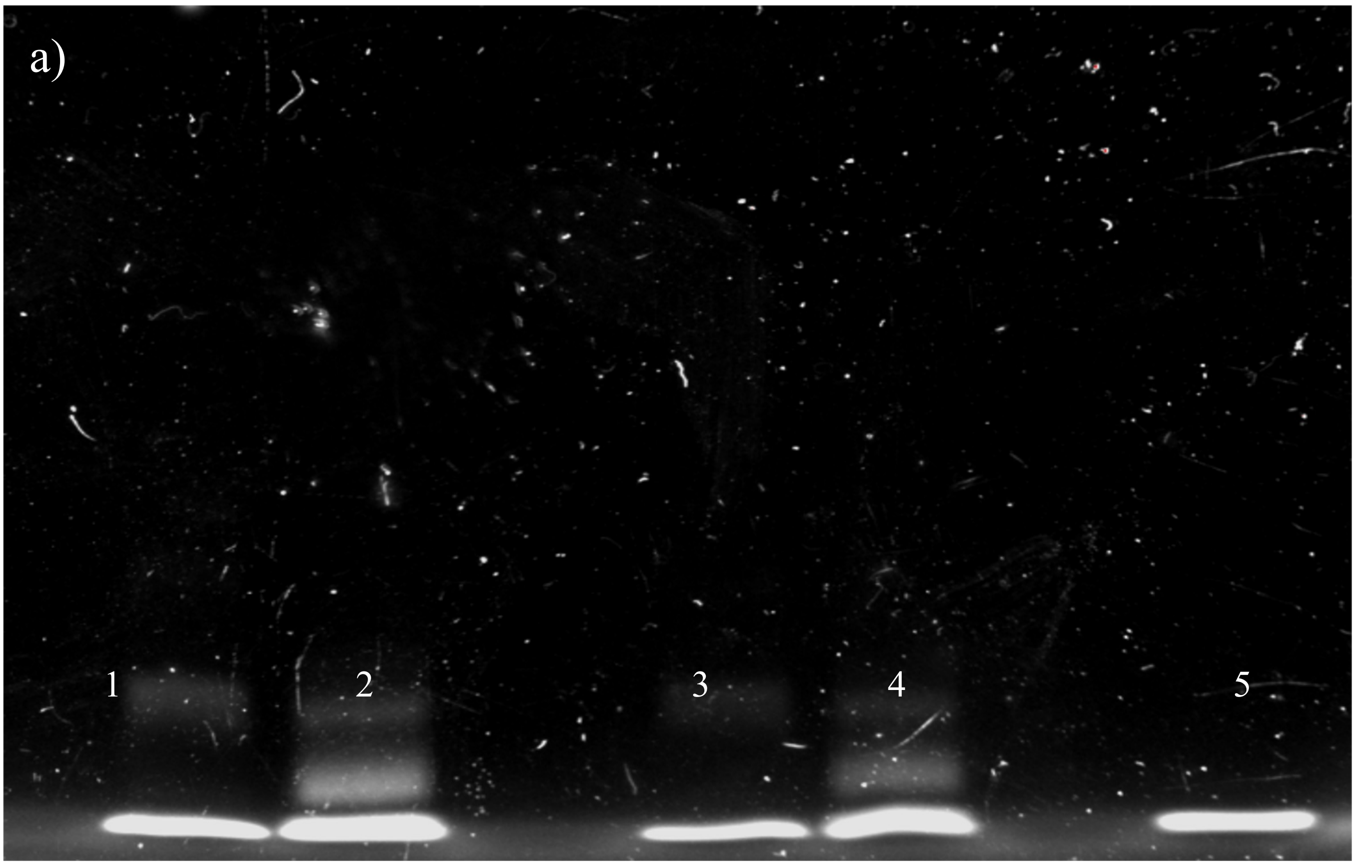

Supplementary Figure 3. A) SDS_PAGE of Lysozyme conjugation to LUVs. 1,3 – LUVs without maleimide group (control liposomes) 2,4 – LUVS with maleimide group (immunoliposomes formulation), (2) lysozyme treated by TR at ratio 1:50 (4) TR ratio 1:20 (4) 5 – monomeric lysozyme

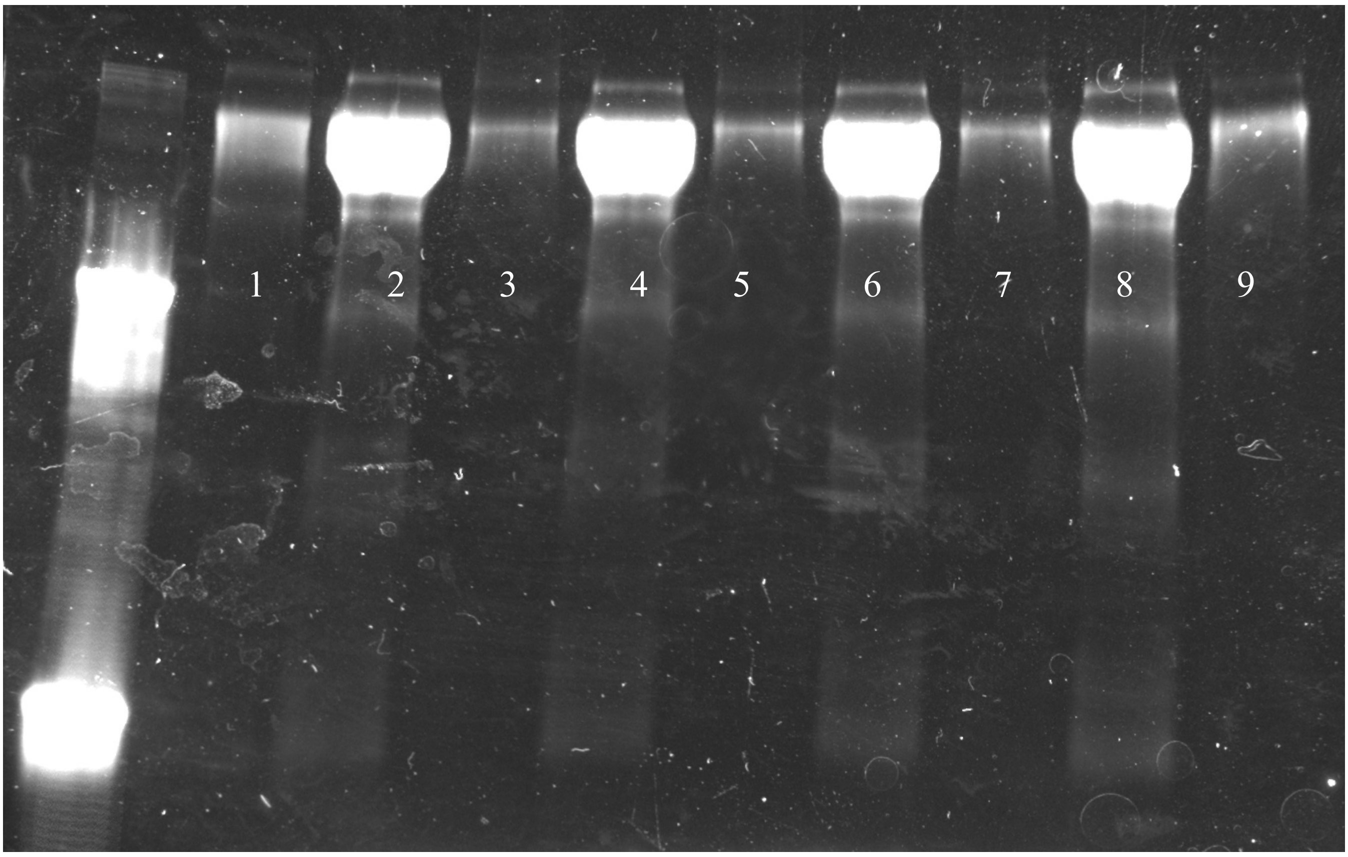

Supplementary Figure 3. B) SDS_PAGE of IgG conjugation to immunoliposome formulation after ultracentrifugation

1 – is monomeric IgG; and 2-9 are supernatant and pellet fractions alternates correspondingly.

2,3 - IgG treated by TR at ratio 1:5; 4,5 – TR ratio 1:10; 6,7 – TR ratio 1:20 and 8,9 – TR ratio 1:50
