# Supplementary material for: Immunoliposomes for detection of rupture-prone intracranial aneurysms
Source: Acta Neurochir (Wien). 2023 Sep 26;165(11):3353–60. doi: 10.1007/s00701-023-05770-9 (PMC10624708; doi:10.1007/s00701-023-05770-9)
Supplement: Supplementary file 2 — (DOCX 15 kb) [file 701_2023_5770_MOESM2_ESM.docx]

Supplement 2

Liposomes consist of the following phospholipids: Dipalmitoyl-phosphotidyl-choline (DPPC), distearoyl-phosphoethanolamine [methoxy (poly-ethylene glycol)-2000] (mPEG2000-DSPE), maleimide derivatized PEG2000-DSPE (mal-PEG2000-DSPE), rhodamine-labeled phosphatidyl dipalmitoyl ethanolamine (Rh-PE), dipalmitoyl-phosphoethanolamine - diethylenetriaminepentaacetic acid (gadolinium salt) (Gd-DTPA-PE) and cholesterol (CHOL). DPPC, CHOL, Rh-PE and Gd-DTPA-PE were purchased from Avanti Polar Lipids. mPEG2000-DSPE and mal-PEG2000-DSPE were purchased from Quanta BioDesign. All lipids were of high (>96%) analytical grade.

**Preparation of liposomes**

Concentrated lipid solutions were prepared by dissolving lipid powder in chloroform and stored in the freezer (-20°C).

Liposomes were prepared using a Hamilton glass syringe to aliquot the corresponding amounts of lipids in a round bottomed flask. The range of molar ratio of lipids were used as follows: DPPC 75%; CHOL 20%; mPEG2000-DSPE 6% without mal-PEG2000-DSPE and 4% with; mal-PEG2000-DSPE 2%; Rh-PE - 0.5%. After mixing desired amounts of lipids, they were dried under nitrogen gas stream at room temperature in the dark until chloroform was evaporated and lipid film was formed. To completely remove traces of chloroform, the lipid films were placed under vacuum for at least 4 hours or overnight. Then, to make multilamellar dispersions, the lipid film was hydrated with 10mM phosphate buffered saline (PBS, pH 7.4) containing 4mM EDTA for 1-2h. The hydration step was performed at the temperature above phase transition of lipids in liposomes. In our lipid formulations, it is determined by presence of DPPC and set in the range 41-50 °C

To produce large unilamellar vesicles (LUVs), the resultant multilamellar solutions were passed 20-25 times through polycarbonate membrane filter with pore sizes of 100 nm in the extruder LiposoFast from Avestin (Canada) under pressure at 40PSI. Extrusion was performed in water bath at temperature set above phase transition, in the range of 41-50°C.

The size and polydispersity of liposomes is assessed by utilizing a Malvern Dynamic Light Scattering Instrument. The instrument was checked with polystyrene beads of 100 nm. The diameter obtained is in the range 70 - 150 nm with polydispersity index varied from 0.02 to 0.15. (Supplementary figure 1. )

Obtained liposomes kept in dark glass vials and stored at 4°C.

**Preparation of immunoliposomes via antibody conjugation**

Conjugation of antibodies to the prepared maleimide containing liposome is based on disulfide modification of protein by Traut’s reagent and formation [-SH] groups on the surface of antibody molecule. Sulfhydryl group, also called “thiol group”, specifically reacts with maleimide group and thus provides the linkage to maleimide moiety at PEG2000 - DSPE in the liposome. The antibody is first thiolated with 2- iminothiolane at a molar ratio of 2-iminothiolane to antibody in the range of 10-50:1 for 1-2h at room temperature (RT). EDTA was included to reaction buffer (PBS, pH7.4) to chelate divalent ions and therefore preserve sulfhydrils from oxidation.

Secondly, the unreacted 2- iminothiolane was removed using a desalting gel column Sephadex G-25 equilibrated by PBS at pH 7.4 and 4mM EDTA buffer. After the gel filtration the parts of conjugated sample was subjected to Ellman’s test to assess number of [-SH] groups formed during thiolation. Briefly, Ellman’s reagent is 5,5’-dithio-bis-(2-nitrobenzoic acid), also known as DTNB reacts with free sulfhydryl groups in solution. The reaction produces a yellow-colored product and can be quantiﬁed by spectrophotometer using an absorbance at 412 nm and extinction coefficient 14150 M^-1^cm^-1^

Lastly, to prepare antibody-linked liposomes (immunoliposomes) the thiolated antibodies were immediately mixed with the PEGylated liposome containing maleimide and incubated 12-18 hours at 4-8 °C. To remove non-linked antibodies the solution was then subjected to an ultracentrifuge and spinned at 100,000g for 1-3 hours. The supernatant was carefully removed. Desired volume of PBS, pH 7.4 and 4mM EDTA added to pellets, and they were rehydrated during at least 3 hours or overnight at 4-6 °C with gentle stirring. Re-dissolved pellet contains immunoliposomes and can be used in further tests.

**Preparation of immunoliposomes containing Gd**

Immunoliposomes with Gd were prepared in two ways. First, lipid molar ratio in the formulations were used as DPPC 75%; CHOL 20%; mPEG2000-DSPE 6% without mal-PEG2000-DSPE and 4% with; mal-PEG2000-DSPE 2%; Rh-PE - 0.5%. Multilamellar vesicles were made by hydration of lipid film with DOTAREM (Guebert, Paris, France) solution with concentration of 280mg/ml. To remove unloaded DOTAREM, LUV obtained after extrusion were subjected to desalting gel column Sephadex G-25 equilibrated by PBS at pH 7.4.

Second way was to introduce Gd-DTPA-PE in lipid formulation. In this case the lipid molar ratio was DPPC 70%; CHOL 20%; mPEG2000-DSPE 6% without mal-PEG2000-DSPE and 4% with; mal-PEG2000-DSPE 2%; Rh-PE - 0.5% and Gd-DTPA-PE 5%. Preparation of LUV containing Gd were the same as described in the section “preparation of liposomes”.

To obtain immunoliposomes by conjugation of corresponding antibodies we employed the same protocol as described in the section “preparation of immunoliposomes”

**Negative control**

For negative control a lipid formulation without maleimide group has been used. Then, corresponding antibody has been added and this mixture underwent same steps of the conjugation protocol described above. Thus, we ensure that antibody in immunoliposomes and mixture of antibody and control liposomes subjected to the same actions.
